# Correction: Comparison of the cardiovascular system, clinical condition, and laboratory results in COVID-19 patients with and without vitamin D insufficiency

**DOI:** 10.1186/s12879-022-07915-0

**Published:** 2022-12-09

**Authors:** Erfan Kazemi, Ali Mansoursamaei, Marzieh Rohani-Rasaf, Hossein Sheibani

**Affiliations:** 1 Student Research Committee, School of Medicine, Shahroud University of Medical Sciences, Shahroud, Iran; 2 Clinical Research Development Unit, Imam Hossein Hospital, Shahroud University of Medical Sciences, Imam Ave., Shahroud, 3616911151 Iran; 3 Department of Epidemiology, School of Public Health, Shahroud University of Medical Sciences, Shahroud, Iran

**Correction: BMC Infectious Diseases (2022) 22:441** https://doi.org/10.1186/s12879-022-07438-8

The original publication of this article [1] contained an incorrect author name. The incorrect and correct information is available in this correction article. The original article has been updated.

Incorrect: Ali Mansursamaei.

Correct: Ali Mansoursamaei.
